# Medical Items and News of Interest to the Physician

**Published:** 1871-07

**Authors:** 


### Medical Items and News,

### Of Interest to the Profession.

CULLED FROM THE STANDARD MEDICAL JOURNALS OF THE WORLD.

NOTE.—These formulæ are intended only for the regular practitioner of medicine, and should be employed only by him, or under his immediate supervision.

JOHN OPPOLZER, the famous Surgeon of Vienna, is dead.

**Nausea following Opium.**

Dr. J. Y. Dale, of Lemont, Pa., (*Georgia Med. Companion*), finds that bromide of potassium will prevent nausea following the use of opium.

**Acute Rheumatism.**

Dr. J. T. Davis, Laconia, Ind., (*Cin. Med. Repository*), asserts that rheumatism is successfully managed in the West by use of quinia sulphas, as a base of treatment.

**Graduates in Medicine.**

The number of medical graduates in the different Medical Colleges of the United States, for 1871, amounts to *one thousand and fifty*.—Starvation and death!

**Dilution of Lymph.**

Dr. J. P. Baker, in a communication to the *British Med. Journal*, on Vaccination, observes that the virus can be preserved in glycerine, and that it may be diluted much more than ten times without perceptible loss of energy.

**A New Professor.**

D. Hayes Agnew, M. D., was *unanimously* elected to the Chair of Principles and Practice of Surgery, made vacant by the resignation of Prof. H. H. Smith, at the University of Pennsylvania, on the 15th of May.

**Diphtheria.**

Dr. C. A. Rothe, (*Berliner Klin. Woch.*), applies carbolic acid to the seat of disease by means of a hair pencil. The patient is to use, at the same time, as a gargle, ten or fifteen drops of this agent to a tumbler of water.

**Dr. Brown-Sequard**

Has been driven from Paris by the insurrection, and taken up his permanent residence in Boston. He is a "high-priced" man, having charged a gentleman, in this vicinity, fifteen hundred dollars to inform him that he had a 'boil in his ear."

**Gastric Douche.**

An apparatus for applying the douche to the stomach is described by Dr. Ploss, of Leipsic. It is indicated in chronic gastric catarrh, poisoning, pyloric stricture, and pyrosis. It has a double channel, but resembles the nasal douche in principle.—*Ibid.*

**Remedy for Smallpox.**

Dr. Steimer says that the muriate of quinine acts as quickly and surely in smallpox as in intermittent fever, and makes vaccination useless. In the stage of eruption, three grains are given every two hours; the fever and even the pustules disappear slowly from the tenth to the twelfth hour. At a later stage, restoration needs from three to five days.—*Berlin Allgem. Centr. Zeitung.*

**Velpeau's Mixture.**

The subjoined formula is the original Velpeau's Mixture:

℞

Tinct. Opii.

Tinct. Camph. Com.

Tinct. Rhei, āā ℥ j.;

Ess. menth. pip. ʒ x.;

Tinc. capsica, ʒ vj. M.

—*Medical Record.*

**Enlarged Tonsils.**

Dr. James Martin states (*Ibid.*), that an eminent Dublin practitioner finds the sulphate of potassa, administered daily for a month or six weeks, almost a specific for enlarged tonsils in children. From five to fifteen grains are given every morning, with a small quantity of rhubarb and aromatics. The dose should produce some laxity of the bowels, and must be diminished if it causes purging.

**Case of Obscure Cerebral Disease.**

J. Cummiskey, M. D., Physician to St. Mary's Hospital, Philadelphia, in a communication to the Phila. *Med. Times*, relates a case of a German girl æt nineteen, who entered the Hospital with symptoms of acute meningitis. For some days she was very violent, and difficult to control; finally became calmer, then passed into a condition of apparent imbecility. She would sit day after day in the same position, noticing nothing that was passing around her, nor replying to questions. She was given ten grains of *iodide of potassium* three times daily. She was discharged entirely cured in three weeks from the time of the administration of the first dose.

**Cæsarian Section.**

In an interesting article upon this subject, by Dr. C. F. Redenstein, in the *Am. Jour. of Obstetrics*, the following conclusions are drawn: 1st. The introduction of sutures is positively indicated when the uterine wound is not closed by the contraction of that organ. 2d. The closing of the uterine wound by sutures does not interfere with the success of the operation. 3d. The application of uterine sutures after every Cæsarian section will probably diminish the rate of mortality attending that operation.

**Pay of Navy Surgeons.**

The following is the pay established by Congress for Assistant Surgeons, and Surgeons, approved July 15th, 1870:

The Fleet Surgeon's pay is $4,400. Full Surgeon, while on duty at sea, for first five years, $2,800; for second five years, $3,200; for third five years, $3,500; fourth five years, $3,700; for twenty years and upwards, $4,200. Passed Assistant Surgeons receive $2,000; Assistant Sugeons, $1,700, while on duty.

**Infantile Epilepsy.**

Dr. F. K. Bailey, of Knoxville, Tenn., through *Med. and Surg. Reporter*, recommends chloral in infantile epilepsy. The following is his formula:

℞

Chloral Hydrate, ʒ j;

Syrp. simp., ℥ jss;

Spts. æthris comp., ʒ j;

Syrp. zingiberis, ʒ iij.

M.

Sig:—½ teaspoonful ter in die.

**Idiopathic Anæmia.**

Prof. Samuel G. Armor, at one of his medical clinics, as reported in the *Med. & Surg. Reporter*, recommended, first, good nourishing food, moderate out-door exercise, and agreeable mental and moral surroundings. In addition to this, a good blood and nerve tonic, as follows:

℞

Iron by hydrogen, ʒ jss;

Quin. sulph., ʒ j;

Arsen. acid, gr. j. M.

M. fiat pil. No. 50.

Sig:—One after each meal.

If constipated, he also recommends, in addition, a pill of one-half grain each of watery extract of aloes and hyocyamus; one to be taken after dinner to keep up gentle eliminative action along the intestinal track.

**Incontinance of Urine.**

Dr. Bradbury, of Aldenbrook's Hospital, Cambridge, relates the successful treatment of a case of incontinance of urine in a girl, (*British Med. Journal,*) who had been so troubled for nine years, and who was cured with one dose, of fifteen grains, of chloral hydrate. She was ordered this every night, but after the first dose there was no return of the complaint.

---

**Ozone in Phthisis Pulmonalis.**

Dr. Dewees relates a case of pulmonary phthisis of four years standing, which seemed much relieved by the daily inhalation of ozone, while simple oxygen failed to produce the same effect. The doctor thought the ozone would prove especially serviceable, by its disinfecting action, in the "putrescent forms" of phthisis. In a case of asthma, complicated with renal disease, similar benefit was obtained.—*Med. Record.*

---

**American Medical Association.**

The Association, at the late meeting held in San Francisco, Cal., awarded the first prize to Dr. P. R. Taylor, of Sacramento, for an essay on the "Chemical Constitution of Bile." The second prize to Dr. Benj. Howard, of New York, for "A Direct Method of Artificial Respiration for the Treatment of Persons Apparently Dead from Suffocation by Drowning, or from Other Causes." Dr. D. W. Yandell, of Kentucky, was elected President for the ensuing year. Dr. Henry A. Martin, of Boston, Chairman of the Committee on Vaccination, was removed for alleged "insulting language towards the Association, in an article on vaccination, which he had published in a homœpathic journal.

---

**Strychnia in Typhoid Fever.**

Dr. N. S. Davis, of Chicago, (*Chicago Med. Examiner*), when the muscular contraction of the heart becomes weak, and the sphincter muscles give indication of relaxation in the advanced stage of typhoid fever, resorts to the use of strychnia. He gives it with nitric acid, and when the bowels move frequently, tincture of opium is added. This is his formula:

℞

Strychnia, gr. j;

Nitric acid, ʒ j;

Tinct. opii, ʒ iij;

Water, f. ℥ iij. M.

Sig:—Give one teaspoonful every four hours, and the turpentine and laudanum emulsion to be given between.

**Chronic Aural Catarrh.**

Dr. James Collins recommends syringing the ear with tepid water, in which has been dissolved five grains of *zinci sulpho-carbolate* in ℥ ij. of water. Correct the nutrition of the patient by the following:

℞

Potass. iodide., ʒ ij;

Tinct. cinch. co., f. ℥ j;

Ext. Stillingia, f. ℥ j;

Aquæ, f. ℥ j. M.

Sig:—Teaspoonful twice daily, after meals.

---

**Tenesmus.**

Dr. G. Kaiser has used, with much benefit, the vapor of chloroform to relieve the tenesmus of acute and chronic dysentary. He puts a drachm of chloroform in a three-ounce bottle, attaching over the neck a rubber or metallic canula. The canula is inserted in the rectum, and the bottle placed in warm water until the chloroform has evaporated. This is to be repeated two or three times a day until the tenesmus subsides.—*Nashville Jour. Med. and Surg.*

---

**New Material for Suppositories.**

Mr. T. Carre, of Meaford, has communicated to the *Canadian Pharmaceutical Journal* a formula for a new material for the administration of opium or other medicine by suppository. He says that in trials made upon patients suffering from hemorrhoids he has found it, from its elastic texture, to possess advantages over other excipients used for a like purpose. Gelatine being one of the ingredients, it could not be used for making suppositories containing tannin, as an insoluble and inert substance would be formed. The following is the formula:

Best glue, ℥ iv;
Glycerine, ℥ viij;
Golden Syrup, ℥ ij;
Water, ℥ viij.

Soak the glue in the water until quite soft, then dissolve over steam or water-bath. Mix the syrup and glycerine well together, add them to the glue solution, and boil until they lose about 2 oz. in weight; then pour out on an oiled tray, or into any suitable mould, previously removing any scum formed. The result is an elastic substance, which will keep for a long time, but dissolving more readily when fresh. When required for use, the composition should be dissolved in a little water with gentle heat, the opium or other drug mixed with it, and then run into a mould.

**Acne.**

Dr. Morris, before the Baltimore Medical Association, recommended the following local application in acne vulgaris:

℞.

Sulphur,

Glycerinæ, āā ʒ vj;

Alcohol., f. ℥ vj.

M.

For Acne. Rocacea he recommended the following formula:

℞.

Potass. acet.,

Glycr., āā. ʒ vj;

Alcohol., f. ℥ vj.

M.

---

**Poison Oak.**

Dr. Jas. S. Bailey, of New York, recommends the following formula for poison induced by the poison oak:

℞.

Hydr. bi. chlor., ʒ ss;

Distilled water, f. ℥ iij;

Add and dissolve

Mur. ammonia, ʒ j;

Nitrate potassa, ʒ ij.

M.

Sig:—Apply thoroughly three times per day with a camel's hair pencil until the parts affected are inflamed in consequence, then discontinue for a few days, using instead calomel ointment, U. S. P., until the parts are healed.—*Med. and Surg. Reporter.*

---

**Mammary Abscess.**

Dr. Jos. R. Beck, of Lancaster, O., writes an interesting article to the Phila. *Med. Times*, upon this subject. He recommends that when the well known symptoms of inflammation of the gland present themselves, every effort should be made to arrest the secretion of milk. The treatment must be begun at once. For this purpose he recommends the following:

℞

Ext. Belladon. Alcoholic., ʒ iv;

Glycerinæ, q. s.;

Mix them to the consistence of a moderately thin paste. This is to be spread in a medium thick layer with a spatula, over and upon both mammary glands, from the sternum to the axilla. Cover with a cloth dipped in olive oil, and this in turn with oiled silk. This he allows to remain from two to four weeks. He adds:

"The argument in the case is directed, of course, to threatening abscesses; but all will at once recognize the appropriateness of the treatment in cases of still-born children, where it is certainly desirable to arrest the secretion of milk at once. In these cases apply the remedy within an hour or two after the birth of the child. I have never known this treatment to fail of its desired effects, where it was used in time."

---

**Turkish Bath in Albuminuria.**

E. Morris, M. D., (*Brit. Med. Jour.*), says that his experience in the treatment of Bright's Disease is, that no medical agent we employ has so great power of averting the complaint, or of prolonging life as the Turkish bath, during the progress of this intractable disease of the kidneys. The only objection raised against its use by the patient is the length of time required for a complete bath—at least two hours being necessary for this process; and when this has to be repeated two and even three times during the day, it becomes a heavy tax upon means and shortens business hours. This objection may, however, be overcome in some measure, by taking the bath early in the morning and the last thing at night.—*Med. Record.*

---

**Pertussis.**

Dr. J. Caldwell, of Brooklyn, recommends, through the *Boston Med. & Surg. Journal*, the use of the Spray Atomizer with the following solution:

℞

Ext. belladon. fld. gtts. v. to x.;

Potass. bromid., ℈ j;

Ammon. bromid., ℈ ij;

Aquæ dist. f. ℥ ij;

M. ft. solutio.

Of this use a tablespoonful at each application of the atomizer.

Another—by Dr. Taneyhill:

℞

Syrup ferri iod., f. ℥ iss;

Syrup pruni virgin., f. ℥ ijss.

Sig:—From one-half to one teaspoonful every third hour, to a child fifteen months old.

---

**Fistula in Ano.**

Dr. Edward C. Huse, of Rockford, Ill., writes to the *Medical Record* of a means for curing this difficulty without the aid of the knife. He tells his process substantially as follows: After exploring the fistula with a very small tube and so determining its course and extent, the patient is to be placed in a good light and a glass rectal speculum introduced, with its fenestrum opposite the internal orifice of the fistula. He then takes an ordinary hypodermic syringe with small silver canula attached. The canula is now bent to the required curvature and introduced, when the syringe, filled with tepid water, is screwed on and the surface thoroughly cleansed of all extraneous matter. Next, by pressure, the fistula in its whole extent, should be dried out. Introduce into the speculum a quantity of carded cotton, in order to absorb any of the injection about to be made, so that it may not pass *through* and injure the mucus membrane. Now, re-insert the canula, fill the syringe with the *saturated ethereal tincture* of iodine, attach it to the canula and inject slowly, at the same time gradually withdraw the canula. In this manner every part of the fistulous tract is reached, and readily heals. The operation is not very painful and should be premised with a cathartic and followed with a full anodyne.

---

**Elixir of Calisaya and Iron.**

The *Druggists' Circular* gives the following formula for the manufacture of this popular tonic:

℞
Calisaya bark in powder, ℥ iv;
Cinnamon water, o. ij;
Caraway water, o. j;
Tincture of orange peel,
Alcohol, āā o. ss;
Brandy, o. ij;
Syrup, o. iij;
Soluble pyrophosphate of iron, ℥ ij.

Mix the cinnamon and caraway waters with the tincture of orange peel, and percolate the bark with the mixture. Dissolve the pyrophosphate of iron in the percolate, add the other ingredients, and filter. This contains about one grain of pyrophosphate of iron, and two grains of cinchona bark to a drachm.

---

**Epilepsy.**

Dr. Samuel E. Raine, of White Haven, Tenn., writes to the Phila. *Med. & Surg. Reporter*, that he had a case of epilepsy—a stout young man æt 19. He received a blow on the head when eight years old—when nine years old was taken with epilepsy—paroxysms occurring at intervals from two to four weeks. Has dyspepsia attended with constipation. He concluded epilepsy was caused by indigestion, therefore prescribed the following pill:

℞
Nux vomica, gr. xxx;
Soc. aloes, ʒ j;
Pulv. Rhei.;
Pulv ipecac., āā gr. x. M.

Ft. pil. No. 40. Sig:—One every morning, and increase to one twice a day.

He had no more fits for a year and a half, when they returned. He was then treated with tincture of aconite in ten drop doses every two hours, until pulse was reduced to fifty per minute. He was relieved after the second dose.—Aconite continued at intervals of four hours—then, ter die. This treatment again arrested the epilepsy.

